# Increased social deprivation index scores are associated with 180-day readmissions, but not index admissions, for acute heart failure

**DOI:** 10.1371/journal.pone.0327123

**Published:** 2025-07-03

**Authors:** Robert R. Ehrman, Brian D. Haber, Nicholas E. Harrison, Steven J. Korzeniewski, Lindsay Maguire, Samantha D. Bauer, Phillip D. Levy

**Affiliations:** 1 Department of Emergency Medicine, Wayne State University School of Medicine, Integrative Biosciences Center, Detroit, Michigan, United States of America; 2 Department of Emergency Medicine, Indiana University School of Medicine, Indianapolis, Indiana, United States of America; 3 Department of Emergency Medicine, University of Kansas Medical Center, Kansas City, Kansas, United States of America; 4 Department of Family Medicine and Public Health Science, Wayne State University School of Medicine, Integrative Biosciences Center, Detroit, Michigan, United States of America; Cleveland Clinic, UNITED STATES OF AMERICA

## Abstract

**Purpose:**

Hospital readmissions are a pervasive problem for patients with heart failure. While Social Determinants of Health (SDoH) influence many aspects of care, the relationship between readmissions for acute heart failure (AHF) and social vulnerability is incompletely characterized. Such data are needed to develop interventions to maximize successful stabilization in the post-discharge phase.

**Methods:**

Retrospective review of administrative clinical data paired with ZIP code-level SDoH data from an integrated health system in Detroit, MI. We explored the relationship between Social Deprivation Index (SDI; greater scores indicate more deprivation) and hospital admissions for AHF within 180-days of a prior AHF admission using zero-hurdle regression (logistic model for >0 readmissions; negative binomial model for count of readmissions). Mixed-effects logistic regression, accounting for repeat visits, was used to determine if SDI was associated with AHF-admission for any given ED visit.

**Results:**

From January 2022 through December 2023, with data from 2,333 unique patients (accounting for 3,281 total visits), we found that each SD increase in SDI (30.6) was associated with increased likelihood of at least one 180day-readmission (OR 1.52 [CI 1.10–2.11]). In the count model, each SD (28.3) increase in SDI was positively associated with 180day-readmissions (relative risk (RR) 1.57 [CI 1.10–1.23]). In the mixed model, after adjusting for characteristics of prior visits, SDI was not associated with AHF admission (including at Index visits).

**Conclusion:**

These results indicate that area-level social vulnerability may play a role in recovery and stabilization after a decompensation event; it may also extend the post-discharge vulnerable phase. That SDI was not associated with Index AHF admission suggests that social factors may play a different role in development of acute decompensation, as opposed to recovery from it. Development of targeted admission-reduction interventions should consider the varied influences of social vulnerability in the AHF lifecycle.

## Introduction

Heart failure (HF) is expected to affect >8 million Americans by 2030, which will cost the United States healthcare system nearly $70 billion [1]. Despite a core research goal from the American Heart Association of improved understanding of how social determinants of health (SDoH) impact HF care [2], the role of social vulnerability in short- and long-term prognosis is incompletely characterized. Of 59 studies assessing the role of SDoH on HF outcomes, a systematic review found that the most-commonly-assessed factors were age, sex, and race/ethnicity; only 8 considered measures of social vulnerability, such as education, poverty, and health literacy [3].

Consideration of SDoH has become a pillar of efforts to improve health equity, yet data from the past 2 decades on how such factors contribute to racial differences in HF outcomes are lacking. Incident HF is greater for Blacks compared to Whites [4], and while overall acute HF (AHF) hospitalizations fell by 31% between 2002 and 2013, the rate for Blacks increased by greater than 200% compared to Whites [5]. HF-related cardiovascular mortality is 1.3-fold greater for Black compared to White males, and 1.6-fold greater for females [6]. Reduced quality of life, increased healthcare costs, and increased all-cause and cardiovascular mortality are sequelae of repeated hospitalizations for AHF [1,7].

While individual SDoH may contribute to differential outcomes, place-based characteristics including schools, retail stores, places of employment, and the physical environment may also play a role. Similarly, area-level social vulnerabilities such as local poverty rate, or living in medically underserved neighborhoods or food deserts may be negatively impactful [8]. To address inequities and improve HF care it is imperative to determine if and how area-level SDoH impact AHF-related healthcare utilization. Therefore, the goal of this study was to assess the relationship between area-level SDoH and AHF readmissions within a single health system in Detroit, MI.

## Methods

### Study design and participants

This retrospective cohort study leverages administrative data collected from January 2022 through December 2023 in an integrated health system located in Detroit, MI that is affiliated with Wayne State University (WSU). Patients selected for inclusion were those with a primary or secondary ED diagnosis of HF who presented to any of the four hospitals in the system. The study was approved by the WSU Institutional Review Board (M1 panel, protocol # IRB-23-08-6019; a waiver of consent was granted according to 45CFR46.116). The administrative data set was accessed on May 9, 2024; patient-identifying information (medical record number) was available, and necessary to track repeat visits by individual patients. Manuscript preparation was in accordance with the Strengthening the Reporting of Observational Studies in Epidemiology (STROBE) guidelines.

### Clinical setting and data

The four hospitals see a combined annual emergency department (ED) volume of approximately 150,000 patients. Included are Detroit Receiving Hospital (DRH), an urban, academic Level 1 Trauma Center; Harper University Hospital (HUH), an urban, academic tertiary care center that does not receive 911 traffic; Sinai-Grace Hospital (SGH), an urban, community teaching hospital; and Huron Valley Sinai Hospital (HVSH), a suburban, academic-affiliated hospital.

Available health system data at the patient-level included: date of service, birthdate, biological sex, ED disposition (admit, discharge, left against medical advice (AMA), and expiration in the ED), ZIP code (from “home/primary” address filed in the electronic medical record, and ICD10 codes assigned for billing purposes. Admission location (ICU, floor etc.) and hospital disposition were not available, nor was ZIP + 4 or patients’ full street address. Unique medical record numbers were available, allowing identification and tracking of repeat visits.

### Case definition

Heart failure was defined by ICD10 code I50.x, but patients were excluded if reason for presentation appeared confounded by a co-occurring condition (e.g., sepsis, pulmonary embolism; see S1Table for full list, n = 187 patients).

We performed chart reviews for a random sample of 50 included, and 50 excluded, patients to confirm the presence or absence of AHF (no erroneous inclusions or exclusions were found during manual adjudication). Patients who died in the ED were excluded (n = 21), as death is outside the scope/focus of this investigation.

#### Primary and secondary exposures.

Area-level data were extracted from WSU’s PHOENIX Virtual Data Warehouse [9]. As our clinical data included home residence ZIP codes without street addresses or ZIP + 4, we chose Social Deprivation Index (SDI) as our primary exposure. SDI is a seven-item composite score that includes: percent living in poverty, percent with < 12 years of education, percent single-parent households, percent living in rented housing units, percent living in overcrowded housing units, percent of households without a car, and percent non-employed adults < 65 years of age; scores range from 1 (least) to 100 (most deprivation) [10].

Secondary area-level exposures available included ZIP-code-level data on the following metrics: Extreme Income Disparities (Index of Concentration at the Extremes, ranging from −1 (least wealth) to 1 (most wealth) [11], source: US census bureau), lack of health insurance (as percentage of adults aged 18–64 years; source Centers for Disease Control and Prevention), and Emergency Department Utilization (as ED visits per adult population, from ED surveillance data), hypertension prevalence (from local ED surveillance data, using ICD10 codes for hypertension), and median systolic blood pressure (SBP, from ED surveillance data). The latter two were chosen given the close link between hypertension and development of HF and progression of disease.

### Primary and secondary outcomes

The primary clinical outcome of interest was occurrence of at least 1 hospital re-admission for AHF within 180-days of a prior AHF-related hospital admission (**Outcome 1**). We hypothesized that increased social vulnerability (greater SDI) would be associated with greater likelihood of 180d-readmission. This 180-day endpoint was chosen to allow sufficient time for SDoH to manifest themselves, as opposed to shorter time scales that may more prominently reflect under-treatment and/or treatment failure at the initial encounter. While the exact timeframe over which SDoH have maximal impact on AHF outcomes is not known, the chosen timepoint extends beyond the early post-hospital discharge vulnerable phase [12,13], when adverse outcomes are greatest for AHF patients. Secondary outcomes included the per-patient count of 180-day AHF readmissions (**Outcome 2**), and overall likelihood of AHF-related hospital admission for any given AHF-related ED visit (**Outcome 3**).

For the purposes of 180-day outcomes, a visit was considered an “Index” visit if there were no prior ED visits or admissions within the preceding 180 days, as prior data has found that patients who survive 6 months after an AHF admission have similar adverse event rates independent of clinical profile and systolic blood pressure classification [12]; visits within the first 180 days of the dataset were thus excluded. Patients with a single visit in the last 180 days of the data set were also excluded from 180-day outcome assessment. Given the aforementioned “Index” definition and duration of the data set, it was possible for single patients to have >1 Index visit and 180d-readmit.

### Statistical analysis

Descriptive data are reported as medians (with inter-quartile range (IQR)), or proportions, as appropriate. To account for excess zeros in the count of 180d-readmissions, outcomes 1 and 2 above were jointly modeled using a two-step zero-hurdle regression. The primary outcome (0 versus ≥1 180d-readmission) was modeled with a logistic model for the zero-hurdle component (i.e., likelihood of ≥1 vs 0 readmissions, **Outcome 1**) and a negative binomial model truncated at 1 (representing ≥1 readmission) for the count portion (**Outcome 2**). Patients with at least one 180-day re-admission during the study period were considered to have met the primary outcome.

Predictors included in all models were age, biologic sex, hospital, and SDI. Additional SDoH variables, listed above, were also considered for inclusion. Two- and three-way interactions, as well as non-linear transformation of continuous predictors (restricted cubic splines) were also considered. Numerical variables were mean-centered and scaled. Variable selection was performed using Akaike’s Information Criteria and likelihood ratio testing for nested models. The same predictors were used in both the zero and count components, with the number of ED visits within 180 days of the Index visit included as an offset in the count model. After final model selection, the following diagnostics were performed on each sub-model: visual inspection of QQ plot, studentized residual plot, calibration plot (for logistic model), and plots of DFFITS/DFBETAS. Multi-collinearity was assessed using variance inflation factor.

Mixed-effects logistic regression was used to model the likelihood of hospital admission for any given AHF-related ED visit. Fixed effects for this model were: age, biologic sex, hospital, days elapsed since prior ED visit (set to 0 for index visits), disposition at prior ED visit (index, admit, discharge, or AMA), and SDI; ZIP code-level factors, as listed above, were also considered. A random intercept was added to account for patient-level revisits. For this model, patients who left AMA were considered as “discharged” in coding the dependent variable. Numerical variables were mean-centered and scaled. Contrasts between levels of categorical variables were calculated as estimated marginal means (i.e., at mean values for continuous variables and averaged across levels of categorical variables).

The distribution of SDI at HVSH was substantially different (see Results), likely due to its suburban location, as opposed to urban location of others. Therefore, sensitivity analyses were performed with patients from HVSH excluded to test whether this altered the association of SDI and outcomes. A ≥ 10% change in the parameter estimate was considered consistent with confounding.

Statistical analysis was performed using RStudio (version 2024.4.2.764), Posit Software, PBC, Boston, MA. Hurdle models were fit using “pscl”, mixed models were fit with “GLMMadaptive”, and marginal means were calculated with “emmeans”. All models were estimated with maximum likelihood and cluster-robust (by hospital) standard errors. Statistical significance was set at 0.05.

## Results

### Participant characteristics

**Fig 1:**
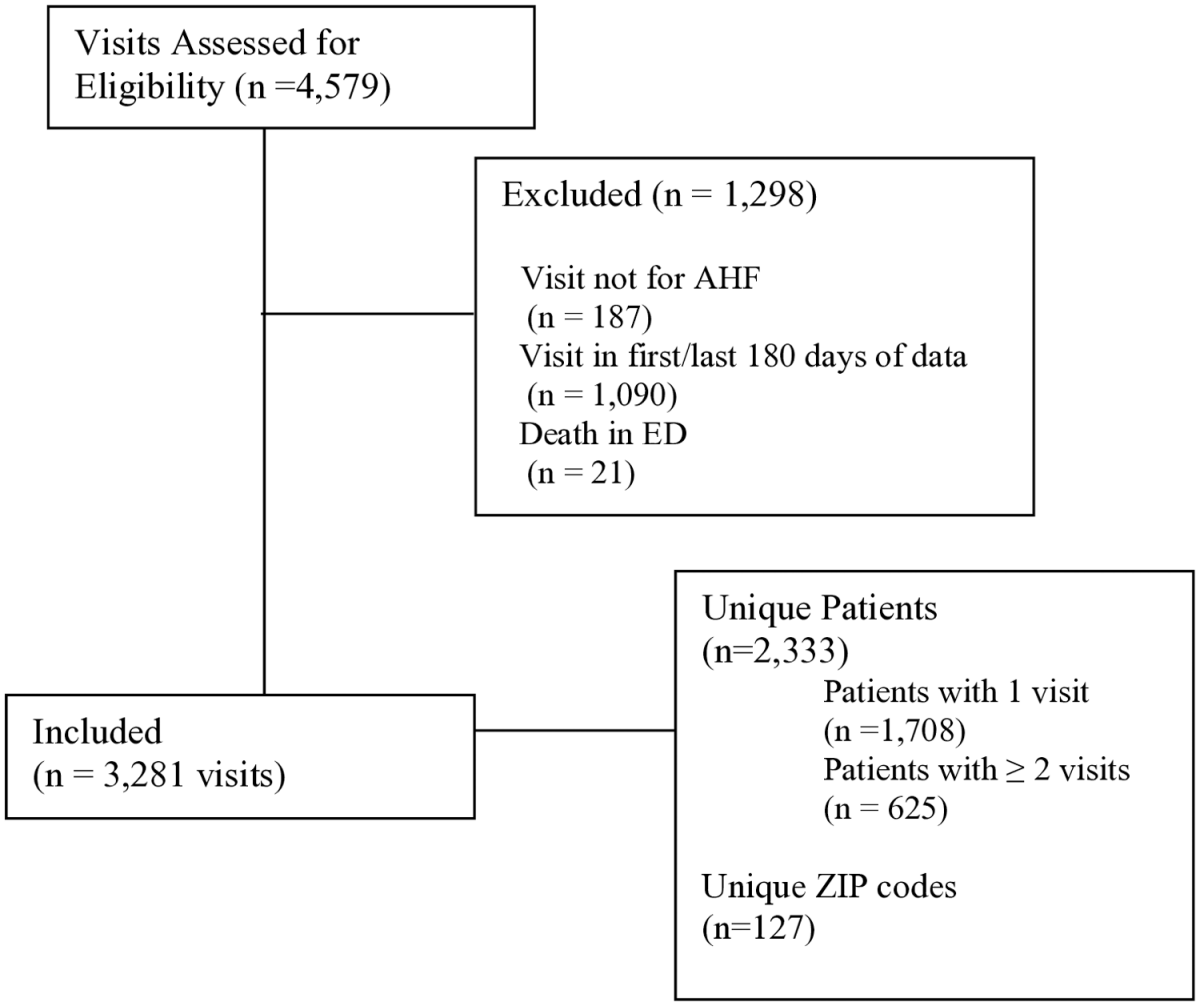
Patient flow diagram.

After exclusions (**Fig 1**), the dataset included 2,333 unique patients residing in 127 ZIP codes. Approximately half the participants were female. For the full cohort, the median age was 66 (IQR 18) years, and the median area-level SDI was 94 (IQR 16). Additional characteristics of unique patients are listed in Table 1.

**Table 1:** Demographics from unique patients.

Characteristic	N = 2,333^1^
Gender	
F	1,131 (48%)
M	1,202 (52%)
Age	66 (18)
Hospital	
DRH	655 (28%)
HUH	589 (25%)
HVSH	398 (17%)
SGH	691 (30%)
Disposition	
Admit	1,867 (80%)
AMA	131 (5.6%)
Discharge	335 (14%)
Total Visits	1 (1)
SDI	94 (16)
Income Disparities	0.18 (0.3)
% Without Health Insurance	11.4 (3.9)
Median SBP	134 (2.0)
HTN Prevalence	47 (10.9)
Days Between Visits	63.7 (129)

^1^n (%); Median (IQR); *F = *female; *M = *male; *DRH = *Detroit; Receiving Hospital; *SGH* = Sinai-Grace Hospital; *HUH* = Harper; University Hospital; *HVSH* = Huron Valley Sinai Hospital; *AMA=*Against Medical Advice; *SDI* = Social Deprivation; Index; *SBP* = systolic blood pressure; *HTN* = hypertension.

### Visit characteristics

The 2,333 unique patients accounted for 3,281 visits (1,708 patients with a single visit and 625 patients with ≥ 2 visits). Overall, the median number of ED visits was 1 (IQR 1) and for patients with >1 visit, the median ED visit count was 2 (IQR 1). The median time between ED visits was 63.7 (IQR 129) days. For patients with at least one 180d-readmission, the median number of re-admissions was 1 (IQR 1); median ED visits for this group was 3 (IQR 2), and median time between ED visits was 47.8 days (IQR 85.8). In patients with no 180-day readmits, median SDI was 94 (IQR 16); for patients with at least 1 re-admission, median SDI was 95 (IQR 5). Median area-level SDI for each hospital was 95 (IQR 2) for DRH, 95 (IQR 5) for HUH, 91(IQR 5) for SGH, and 13.5 (IQR 14) for HVSH.

Hospital admission occurred in 81% of ED visits (2,649/3,281), and 80% of patients (1,867/2,333) had at least 1 admission. There were 2,371 index visits, at which admission occurred in 82% (1,944/2,371). Comparison of disposition on the Index, versus non-Index, visits is shown in Supplemental S2 Table.

### Missing data

Within PHOENIX, SDI was missing for a single ZIP code, resulting in exclusion of 1 visit from primary and secondary outcome analyses. Median SBP was missing for 15 ZIP codes, resulting in exclusion of 17 and 19 visits from the primary and secondary outcome analyses, respectively. Complete data was available for all other variables from PHOENIX and the administrative data.

### Primary and secondary outcomes

A total of 338 patients met criteria for the primary outcome (≥ 1 AHF readmission within 180 days of a prior admission). For the zero-hurdle component (**Outcome 1**), each 1 SD increase in SDI (30.6) was associated with increased likelihood of at least one 180d-readmission (OR 1.52 [CI 1.10–2.11]). In the count model (**Outcome 2**), each SD (28.3) increase in SDI was also positively associated with 180d-readmissions (relative risk (RR) 1.57 [CI 1.10–1.23]).

The final models included age, biological sex, median SBP, hospital, and SDI. Parameter estimates are provided in Table 2, showing significant hospital-level differences, but no significant associations with age, biologic sex, or ZIP-code-level median SBP. Area-level lack of health insurance and income disparities were not statistically associated with readmission and worsened model fit, so they were excluded; lack of health insurance was also colinear with SDI, further supporting exclusion. Magnitudes of association changed by < 10% in sensitivity analyses that removed patient visits to HVSH as a suburban outlier based on low SDI; full model results are provided in the Supplement S3 and S4 Tables.

**Table 2:** Zero-Hurdle negative binomial model.

Characteristic	OR/RR^1^	95% CI^*2*^	p-value
Zero-Hurdle (Logistic) Model			
**Age**	0.96	0.85, 1.08	0.5
**Biological Sex**			
*F*	—	—	
*M*	1.13	0.89, 1.43	0.3
**Median SBP (Zip code level)**	1.06	0.83, 1.35	0.7
**SDI**	1.52	1.10, 2.11	0.011
**Hospital**			
*DRH*	—	—	
*HUH*	1.36	0.98, 1.89	0.067
*HVSH*	2.48	1.10, 5.61	0.029
*SGH*	1.78	1.28, 2.47	<0.001
Count Model			
**Age**	0.93	0.81, 1.06	0.3
**Biological Sex**			
*F*	—	—	
*M*	0.98	0.76, 1.28	>0.9
**Median SBP (Zip code level)**	1.24	0.89, 1.73	0.2
**SDI**	1.57	1.10, 2.23	0.013
**Hospital**			
*DRH*	—	—	
*HUH*	1.12	0.77, 1.64	0.6
*HVSH*	2.58	1.21, 5.50	0.014
*SGH*	1.25	0.84, 1.86	0.3

Continuous Variables are mean-centered and scaled.

^1^Odds-Ratio (OR) for Logistic Model; Relative Risk (RR) for Count Model. ^*2 CI*^ = Confidence Interval; *F = *female; *M = *male; *DRH = *Detroit; Receiving Hospital; *SGH* = Sinai-Grace Hospital; *HUH* = Harper; University Hospital; *HVSH* = Huron Valley Sinai Hospital; *AMA=*Against Medical Advice; *SDI* = Social Deprivation; Index; *SBP* = systolic blood pressure.

### Mixed-effects model (Outcome 3)

Clinical fixed-effects in the final model were age, biological sex, hospital, days elapsed since prior visit, and disposition at prior visit (reference category set to Index visit). Best model fit was achieved with inclusion of SDI and Median SBP, although neither achieved statistical significance (Table 3). Increasing age was associated with greater likelihood of admission (OR 1.24 [CI 1.12–1.36] per SD (14.1 years) increase, as was increasing time between visits (OR 1.19 [CI 1.06–1.33]) per SD (88.8 days) increase (alternatively, OR 1.06 per 29.6 days). Lack of health insurance and income disparities were excluded from the final model for the same reasons as with the primary outcome.

**Table 3:** Odds ratio of admission at any given heart failure-related ED visit.

Characteristic	Odds Ratio	95% CI^1^	p-value
**Age**	1.24	1.12, 1.36	<0.001
**Biological Sex**			
*F*	—	—	
*M*	0.83	0.69, 1.01	0.059
**Hospital**			
*DRH*	—	—	
*HUH*	1.24	0.97, 1.59	0.083
*HVSH*	2.50	1.31, 4.78	0.006
*SGH*	1.39	1.07, 1.81	0.012
**Days Between Visits**	1.19	1.06, 1.33	0.002
**Median SBP (Zip code level)**	1.12	0.94, 1.35	0.2
**Disposition at Prior Visit**			
*Index*	—	—	
*Admit*	1.40	1.07, 1.81	0.012
*AMA*	0.45	0.22, 0.90	0.025
*Discharge*	0.28	0.20, 0.40	<0.001
**SDI**	1.19	0.95, 1.49	0.13
**SD of Random Intercept**	0.28		

Continuous Variables are mean-centered and scaled.

1 ^*CI*^ = Confidence Interval; *F = *female; *M = *male; *DRH = *Detroit; Receiving Hospital; *SGH* = Sinai-Grace Hospital; *HUH* = Harper; University Hospital; *HVSH* = Huron Valley Sinai Hospital; *AMA=*Against Medical Advice; *SDI* = Social Deprivation; Index; *SBP* = systolic blood pressure.

**Fig 2:**
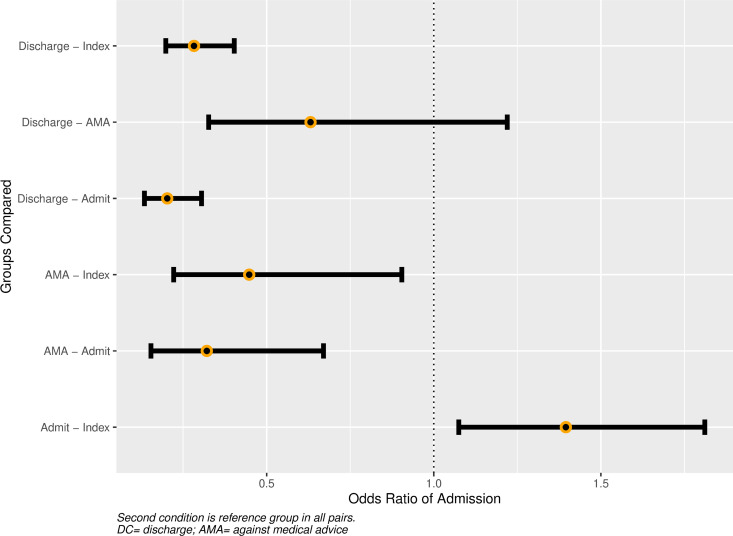
Influence of prior disposition on admission at current Visit. Caption: Second condition is reference group in all pairs. DC = discharge; AMA= against medical advice.

**Fig 3:**
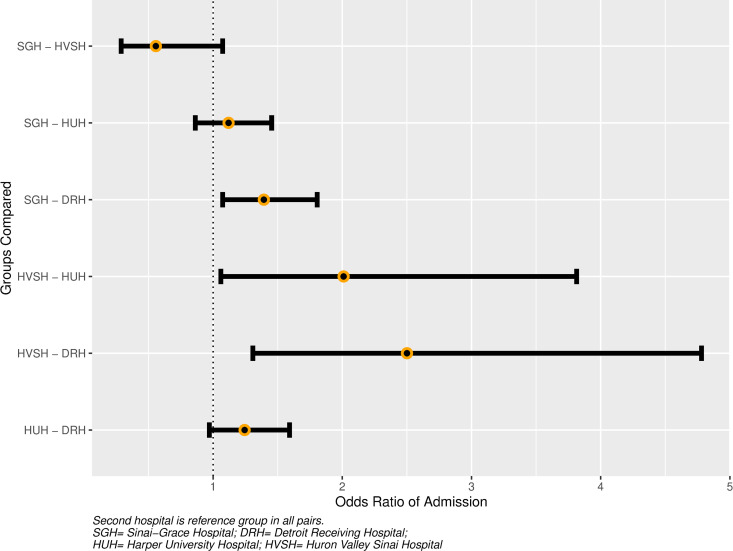
Between-hospital differences in admission. Captio:Second hospital is reference group in all pairs.. SGH = Sinai-Grace Hospital; DR = Detroit Receiving Hospital; HUH = Harper University Hospital; HVSH = Huron Valley Sinai Hospital.

The odds of admission varied by hospital and prior visit disposition, with ORs listed in Table 3 and level-specific contrasts shown in Figs 2 and 3. Compared to Index visits, admission at the subsequent visit was more likely for patients who were admitted at their prior visit (OR 1.40 [CI 1.08–1.81] and less likely for patients who were discharged or left AMA (OR 0.28 [CI 0.20–0.40], and OR 0.45 [CI 0.22–0.90], respectively). Compared to admission at the prior visit, patients who left AMA were less likely to be admitted at the subsequent visit (OR 0.32, [CI 0.15–0.67]). For Index visits alone, the estimated marginal effect on admission for a + 2, versus −2, SD change in SDI was 0.99 (CI 0.83–1.19).

The SD of the random intercept was 0.28, which translates to a patient-level effect on the OR of admission at any given visit of +/-1.73 and indicates substantial patient-level variability, when compared to fixed-effects estimates.

## Discussion

In this retrospective study of AHF patients who presented to a single health system in metropolitan Detroit, MI, increased Social Deprivation Index scores were associated with heightened risk and frequency of 180d-AHF-readmissions, but not Index admissions. These results indicate that area-level social vulnerability contributes to, and potentially extends, the post-discharge vulnerable phase. While we did see variability in associations at the hospital and (unmeasured) patient-level, more work is needed to delineate the mechanisms through which individual and neighborhood-level SDoH influence AHF outcomes.

Prior studies on the relationship between SDoH and AHF outcomes have conflicting results, with some reporting that social disadvantage (e.g., low socioeconomic status, housing instability, low health literacy) is associated with increased hospital readmission and mortality [14–16], while others found no such associations [17–19]. Interestingly, a 2020 study by Patel et al. found relatively small differences in “effect” sizes for 30-day readmission in models that adjusted for age, biological sex, insurance status, and year of index hospitalization (RR = 1.51) compared to those that included these along with HF sub-type and 14 additional clinical variables (RR = 1.45). This suggests that SDoH may be as influential as clinical variables with respect to AHF outcomes [20]. They also reported increased risk of 30-day AHF readmission in increasing quartiles of SDI in Black, versus White, patients. Our study lacked race information, so we were unable to examine racial disparity. Our findings are, nevertheless, likely consistent (in terms of the influence of SDI), given that the population of Detroit predominately identifies as Black (78% according to 2022 census data [21]). Data from our community during the pandemic showing that COVID-19 outcomes were closely tied to social vulnerability, but not race, support this conclusion [22]. Such a consideration is thus of significant interest to the community that we serve, as it provides an area-level measure with components that are modifiable.

While there is overlap in the overarching message of our finding that AHF readmissions are associated with social vulnerability and prior studies, our work differs in several important ways. First, our results suggest that the potential contributions of area-level SDoH may be even broader than recognized when analyzing 30-day outcomes, finding increased readmission incidence even up to 180-days. Reasons for readmission are multifactorial, with clinical and SDoH factors each contributing different time-varying risks. By extending our inclusion period beyond what has previously been reported to be the early vulnerable phase [13,23], we are able to capture the complete time frame when the vast majority of patients are readmitted, reflecting the full range of potential factors that could influence outcomes from physiological to social deprivation. Second, we included novel variables in our model such as median SBP (derived from aggregated and geocoded ED data from an on-going community surveillance project and included in PHOENIX). Poor blood pressure control is common in our community and its prevalence overlaps with social vulnerability [24]. Including this provides a unique measure of community-level factors that could impact the clinical effectiveness of HF and other cardiovascular disease management. Third, we sought to evaluate the contribution of unmeasured patient-level factors and found this led to marked variability in OR of admission (+/- 1.73), a magnitude greater than many of the fixed effects we assessed (Table 3). While this variation is not specifically attributable to SDI (or other social factors), it supports collection of more robust social vulnerability data at the patient level, as composite scores may not serve all patients equally well.

A final aspect in which our study is unique is that we separately explored the influence of area-level deprivation on AHF admissions irrespective of whether it was an Index admission or readmission (while accounting for repeated visits and adjusting for characteristics of the prior visit). We found no association between SDI and overall likelihood of AHF admission for any given ED visit for AHF, including Index visits. This finding is not only novel but raises the question of where to best target resources for reducing AHF disparities. While prior work has demonstrated that early (30–90 days) readmission is a risk for all patients, our results suggest that area-level SDoH may extend this vulnerable period to as far as 180 days. Since Index admissions were not significantly increased by area-level SDoH in our dataset, our findings could mean that they contribute less to *de novo* AHF decompensation, and more to exacerbating patients’ readmission risk after index decompensation. This leads to a troubling potential implication: even if a patient’s ZIP code did not increase the risk of index decompensation, it nevertheless appears to increase the risk of patients in vulnerable neighborhoods developing an admission-readmission spiral once they experience a decompensation event. While beyond the scope of our study to delineate, there exist many plausible reasons this could be true. Area-level social vulnerabilities could be correlates of the distinct, need-specific challenges to prevent readmission: access to follow-up clinics/pharmacies, access to transportation, food security, and healthy dietary options. Other aspects of a patients’ neighborhood could alternatively augment (e.g., safe spaces for physical activity) or impair (e.g., poor air quality) the ability to adhere to a post-discharge regimen of ongoing and intensive disease management. In other words, patients’ neighborhood environment may not be the difference between decompensating overall so much as it prevents patients who have already decompensated from safely transitioning back to a state of well-controlled, medically maximized chronic HF.

Evaluating the specific mechanisms by which neighborhood may influence readmission risk is a critical need for future research. Existing studies on such programs are generally positive [25,26], although more data are needed to delineate the specific pathways through which area-level SDoH may extend or exacerbate the post-discharge vulnerable period. Future studies should focus on the interplay between social and clinical variables, including at the individual level, as next steps to reducing readmission.

### Limitations

Our study has several important limitations. Foremost, it was conducted at a single integrated health system within a confined geographic area, meaning our results may not be generalizable or transportable. We do not have data on patients who presented to hospitals outside our system, and the extent to which this affects our results is not known. We also lack data on length-of-stay at each hospitalization, which alters time at risk for re-admission within the subsequent 180-days; the potential impact of this time-varying confounding on our results is likewise unknown.

Residual confounding is also possible, and like all observational studies, ours cannot distinguish between association and causation. Nor can we rule out the possibility of ecological fallacy with respect to area-level exposures. The few clinical variables available for analysis also restricted our ability to perform further sensitivity analyses or examine racial disparity. Our findings are nevertheless consistent with previous studies that incorporated additional clinical characteristics [20].

Owing to the availability of ZIP code data and not ZIP + 4 or street-level addresses, we were unable to examine geographic boundaries that would be more closely aligned with population characteristics (i.e., census tracts). Further studies, utilizing vulnerability measures from small geographic areas, are needed to confirm our findings. That said, data at the ZIP code-level is generally more widely available, particularly in large databases, and has been meaningfully used on AHF-related predictive models [27]. We used area- rather than individual-level social vulnerability data because it reflects the environment surrounding the patient in the community where they live. While person-level vulnerability characteristics are likely important contributors to outcomes, individual data, when collected, are often inaccurate or incomplete [28,29].

Lastly, patients were identified for inclusion by ICD10 codes from the ED chart, rather than adjudicated final hospital diagnoses. This is a pragmatic approach used in numerous HF studies, and one that has demonstrated excellent positive predictive value [30].

## Conclusion

In this retrospective cohort from four hospitals in a single metropolitan health system, we found that increased social vulnerability was associated with increased 180d-readmissions for AHF, but not Index AHF admissions. Our data add unique insight to the growing body of literature demonstrating a relationship between social vulnerability and AHF outcomes and supports the need for further evaluation of the complex interplay between individual characteristics and area-level exposure to SDoH.

## Supporting information

S1 Table Disposition at index versus non-index visits. (DOCX)

S2 Table Excluded diagnoses not consistent with acute heart failure as primary reason for visit. (DOCX)

S3 Table Negative binomial model without HVSH patients. (DOCX)

S4 Table Odds ratio of admission at any given visit without HVSH patients. (DOCX)
