# Supplementary material for: Increased social deprivation index scores are associated with 180-day readmissions, but not index admissions, for acute heart failure
Source: PLoS One. 2025 Jul 3;20(7):e0327123. doi: 10.1371/journal.pone.0327123 (PMC12225874; doi:10.1371/journal.pone.0327123)
Supplement: S1 Table — (DOCX) [file pone.0327123.s001.docx]

**Table S1:** Excluded diagnoses not consistent with Acute Heart Failure as primary reason for visit

| Diagnosis | n |
| --- | --- |
| Acute bronchospasm | 1 |
| Acute embolism and thombos unsp deep veins of low extrem | 1 |
| Acute kidney failure, unspecified | 24 |
| Acute pancreatitis without necrosis or infection, unspecified | 1 |
| Acute pericarditis, unspecified | 1 |
| Adult failure to thrive | 1 |
| Altered mental status, unspecified | 3 |
| Anemia, unspecified | 4 |
| Bradycardia, unspecified | 1 |
| Cardiac arrest, cause unspecified | 3 |
| Cellulitis of right lower limb | 1 |
| Cerebral infarction, unspecified | 2 |
| Chronic kidney disease, unspecified | 1 |
| Chronic obstructive pulmonary disease w (acute) exacerbation | 59 |
| Disorder of kidney and ureter, unspecified | 1 |
| Dizziness and giddiness | 2 |
| Encephalopathy, unspecified | 1 |
| Encounter for issue of repeat prescription | 2 |
| Epigastric pain | 1 |
| Fracture of unsp part of neck of left femur, init | 1 |
| Gastrointestinal hemorrhage, unspecified | 1 |
| Hb-SS disease with crisis, unspecified | 4 |
| Hypo-osmolality and hyponatremia | 1 |
| Mech compl of other cardiac electronic device, init encntr | 3 |
| Nausea with vomiting, unspecified | 1 |
| Other chest pain, (non-cardiac) | 3 |
| Other pulmonary embolism without acute cor pulmonale | 1 |
| Other specified abnormalities of plasma proteins | 2 |
| Other specified soft tissue disorders | 1 |
| Palpitations | 1 |
| Pleurodynia | 1 |
| Pneumonia, unspecified organism | 19 |
| Poisoning by oth opioids, accidental (unintentional), init | 1 |
| ST elevation (STEMI) myocardial infarction of unsp site | 2 |
| Sepsis, unspecified organism | 6 |
| Syncope and collapse | 8 |
| Tachycardia, unspecified | 2 |
| Transient cerebral ischemic attack, unspecified | 1 |
| Type 2 diabetes mellitus with hyperglycemia | 2 |
| Unspecified abdominal pain | 1 |
| Unspecified asthma with (acute) exacerbation | 6 |
| Unspecified injury of head, initial encounter | 4 |
| Unspecified ovarian cyst, left side | 1 |
| Urinary tract infection, site not specified | 1 |
