# Supplementary material for: Increased social deprivation index scores are associated with 180-day readmissions, but not index admissions, for acute heart failure
Source: PLoS One. 2025 Jul 3;20(7):e0327123. doi: 10.1371/journal.pone.0327123 (PMC12225874; doi:10.1371/journal.pone.0327123)
Supplement: S2 Table — (DOCX) [file pone.0327123.s002.docx]

Table S2: Disposition at Index Versus Non-Index Visits

|  |  | **Disposition at Prior Visit***^1^* | | |
| --- | --- | --- | --- | --- |
|  | **Index Visit***^2^* | **Admit** N = 643*^2^* | **AMA** N = 72*^2^* | **Discharge** N = 195*^2^* |
| **Disposition at Current Visit** |  |  |  |  |
| *Admit* | 1,944 (82%) | 556 (86%) | 42 (58%) | 107 (55%) |
| *AMA* | 133 (5.6%) | 32 (5.0%) | 16 (22%) | 15 (7.7%) |
| *Discharge* | 294 (12%) | 55 (8.6%) | 14 (19%) | 73 (37%) |
| *^1^*The most recent visit compared to current visit | | | | |
| *^2^*n (%)  Rows represent disposition at the most current visit; columns represent disposition at the  Immediate *prior visit,* except “Index”, which by definition, has no prior visit (e.g., of the 72  patients who left “AMA” at prior visits, 42 were admitted at the current visit, 14 were discharged, etc). | | | | |
