# Supplementary material for: Increased social deprivation index scores are associated with 180-day readmissions, but not index admissions, for acute heart failure
Source: PLoS One. 2025 Jul 3;20(7):e0327123. doi: 10.1371/journal.pone.0327123 (PMC12225874; doi:10.1371/journal.pone.0327123)
Supplement: S3 Table — (DOCX) [file pone.0327123.s003.docx]

Table S3: Zero-Hurdle Negative Binomial Model Without HVSH Patients

| **Characteristic** | **OR/RR***^1^* | **95% CI***^2^* | **p-value** |
| --- | --- | --- | --- |
| Zero-Hurdle (Logistic) Model | | | |
| **Age** | 0.97 | 0.86, 1.10 | 0.6 |
| **Biological Sex** |  |  |  |
| *F* | — | — |  |
| *M* | 1.16 | 0.90, 1.49 | 0.3 |
| **Median SBP (Zip code level)** | 1.04 | 0.88, 1.23 | 0.6 |
| **SDI** | 1.42 | 1.16, 1.75 | <0.001 |
| **Hospital** |  |  |  |
| *DRH* | — | — |  |
| *HUH* | 1.40 | 1.01, 1.95 | 0.046 |
| *SGH* | 1.88 | 1.35, 2.63 | <0.001 |
| Count Model | | | |
| **Age** | 0.94 | 0.82, 1.08 | 0.4 |
| **Biological Sex** |  |  |  |
| *F* | — | — |  |
| *M* | 0.86 | 0.64, 1.14 | 0.3 |
| **Median SBP (Zip code level)** | 1.02 | 0.81, 1.28 | 0.9 |
| **SDI** | 1.43 | 0.98, 2.11 | 0.066 |
| **Hospital** |  |  |  |
| *DRH* | — | — |  |
| *HUH* | 1.09 | 0.75, 1.60 | 0.6 |
| *SGH* | 1.20 | 0.79, 1.81 | 0.4 |
| Continuous Variables are mean-centered and scaled | | | |
| *^1^*Odds-Ratio (OR) for Logistic Model; Relative Risk (RR) for Count Model | | | |
| *^2^*CI = Confidence Interval  *F=*female; *M=*male; *DRH=*Detroit  Receiving Hospital; *SGH*= Sinai-Grace Hospital; *HUH*= Harper  University Hospital; *HVSH*= Huron Valley Sinai Hospital;  *AMA=*Against Medical Advice; *SDI*= Social Deprivation  Index; *SBP*=systolic blood pressure; *HTN*= hypertension | | | |
