# Supplementary material for: Increased social deprivation index scores are associated with 180-day readmissions, but not index admissions, for acute heart failure
Source: PLoS One. 2025 Jul 3;20(7):e0327123. doi: 10.1371/journal.pone.0327123 (PMC12225874; doi:10.1371/journal.pone.0327123)
Supplement: S4 Table — (DOCX) [file pone.0327123.s004.docx]

Table S4: Odds Ratio of Admission at Any Given Visit Without HVSH Patients

| **Characteristic** | **Odds Ratio** | **95% CI***^1^* | **p-value** |
| --- | --- | --- | --- |
| **Age** | 1.25 | 1.13, 1.39 | <0.001 |
| **Biological Sex** |  |  |  |
| *F* | — | — |  |
| *M* | 0.84 | 0.68, 1.03 | 0.089 |
| **Hospital** |  |  |  |
| *DRH* | — | — |  |
| *HUH* | 1.25 | 0.97, 1.61 | 0.085 |
| *SGH* | 1.41 | 1.09, 1.83 | 0.009 |
| **Days Between Visits** | 1.18 | 1.05, 1.33 | 0.005 |
| **Median SBP (Zip code level)** | 1.12 | 0.92, 1.35 | 0.3 |
| **Disposition at Prior Visit** |  |  |  |
| *Index* | — | — |  |
| *Admit* | 1.53 | 1.15, 2.03 | 0.003 |
| *AMA* | 0.51 | 0.28, 0.92 | 0.025 |
| *Discharge* | 0.28 | 0.20, 0.40 | <0.001 |
| **SDI** | 1.19 | 0.92, 1.53 | 0.2 |
| **SD of Random Intercept** | 0.42 |  |  |
| Continuous Variables are mean-centered and scaled | | | |
| *^1^*CI = Confidence Interval  *F=*female; *M=*male; *SD=* standard deviation; *DRH=*Detroit  Receiving Hospital; *SGH*= Sinai-Grace Hospital; *HUH*= Harper  University Hospital; *HVSH*= Huron Valley Sinai Hospital;  *AMA=*Against Medical Advice; *SDI*= Social Deprivation  Index; *SBP*=systolic blood pressure; *HTN*= hypertension | | | |
